# Dynamic monitoring soft tissue healing via visualized Gd-crosslinked double network MRI microspheres

**DOI:** 10.1186/s12951-024-02549-7

**Published:** 2024-05-27

**Authors:** Tongtong Chen, Zhengwei Cai, Xinxin Zhao, Gang Wei, Hanqi Wang, Tingting Bo, Yan Zhou, Wenguo Cui, Yong Lu

**Affiliations:** 1 Department of Radiology, School of Medicine, Ruijin Hospital, Shanghai Jiao Tong University, 197 Ruijin 2nd Road, Shanghai, 200025 P. R. China; 2 Department of Orthopaedics, Shanghai Institute of Traumatology and Orthopaedics, Ruijin Hospital, Shanghai Jiao Tong University School of Medicine, 197 Ruijin 2nd Road, Shanghai, 200025 P. R. China; 3 Department of Radiology, Ren Ji Hospital, School of Medicine, Shanghai Jiao Tong University, No. 160, Pujian Road, Shanghai, 200127 P. R. China; 4 Department of Endocrine and Metabolic Diseases, Shanghai Institute of Endocrine and Metabolic Diseases, Ruijin Hospital, Shanghai Jiao Tong University School of Medicine, Shanghai, 200025 P. R. China; 5 Clinical Neuroscience Center, Ruijin Hospital Luwan Branch, Shanghai Jiao Tong University School of Medicine, Shanghai, 20025 P. R. China

**Keywords:** Magnetic resonance imaging, Tissue regeneration, Hydrogel microspheres, Real-time monitoring

## Abstract

By integrating magnetic resonance-visible components with scaffold materials, hydrogel microspheres (HMs) become visible under magnetic resonance imaging(MRI), allowing for non-invasive, continuous, and dynamic monitoring of the distribution, degradation, and relationship of the HMs with local tissues. However, when these visualization components are physically blended into the HMs, it reduces their relaxation rate and specificity under MRI, weakening the efficacy of real-time dynamic monitoring. To achieve MRI-guided in vivo monitoring of HMs with tissue repair functionality, we utilized airflow control and photo-crosslinking methods to prepare alginate-gelatin-based dual-network hydrogel microspheres (G-AlgMA HMs) using gadolinium ions (Gd (III)), a paramagnetic MRI contrast agent, as the crosslinker. When the network of G-AlgMA HMs degrades, the cleavage of covalent bonds causes the release of Gd (III), continuously altering the arrangement and movement characteristics of surrounding water molecules. This change in local transverse and longitudinal relaxation times results in variations in MRI signal values, thus enabling MRI-guided in vivo monitoring of the HMs. Additionally, in vivo data show that the degradation and release of polypeptide (K_2_ (SL)_6_ K_2_ (KK)) from G-AlgMA HMs promote local vascular regeneration and soft tissue repair. Overall, G-AlgMA HMs enable non-invasive, dynamic in vivo monitoring of biomaterial degradation and tissue regeneration through MRI, which is significant for understanding material degradation mechanisms, evaluating biocompatibility, and optimizing material design.

**Supplementary Information:**

The online version contains supplementary material available at 10.1186/s12951-024-02549-7.

## Introduction

Tissue repair and regeneration is a continuous and dynamic process, encompassing cell proliferation, tissue shaping, and ultimately the formation of functional organs, a process that takes a considerable amount of time [1]. Scaffold materials offer a site for cell proliferation, migration, and functional metabolism, facilitating the transmission of cells, signal molecules, and nutrients, as well as waste excretion [2]. The degradation rate of scaffold materials in tissue repair directly affects their efficacy: too rapid degradation leads to insufficient cell adhesion and retention, while too slow degradation inhibits cell migration and differentiation, leading to local fibrosis [3]. Therefore, dynamic monitoring of the entire process is particularly important for optimizing the design of biomaterials and ensuring the effectiveness of tissue repair [4]. However, it has not been possible to achieve real-time non-invasive monitoring of the status of biomaterials in the body. Traditional methods such as histological staining, require euthanizing subjects at fixed time points, precluding continuous observation of the same subject [5]. Recently, non-invasive imaging technologies, such as X-ray computed tomography (CT), ultrasound imaging, magnetic resonance imaging (MRI), near-infrared (NIR), and fluorescence optical imaging (FOI), have seen rapid advancements [6]. Yet, few studies have directly integrated imaging elements into scaffold materials to achieve simultaneous non-invasive, real-time monitoring of scaffold degradation and tissue repair [7]. Therefore, by coupling tissue repair scaffold materials with imaging components, non-invasive real-time monitoring of in vivo status could be achieved [8].

Owing to their superior biocompatibility and tunable adaptability to tissue microenvironments, hydrogels are promising as scaffolds for tissue repair [9]. However, for optimal tissue repair, hydrogel scaffolds must have a degradation rate that aligns with neotissue regeneration [10]. Thus, to ensure sustained and stable repair, and to facilitate future clinical applications, understanding the post-implantation behavior of materials in vivo is crucial [11]. Currently, hydrogel materials have been traced noninvasively by FOI, NIR, carbon nanodot modification, or ^19^F nuclide imaging [12]. For instance, Zhang et al. implanted fluorescently labeled hyaluronic acid hydrogels into mice spinal cords, using fluorescence intensity to gauge in vivo degradation [13]. However, fluorescence monitoring, prone to diffuse signals, failed to precisely depict the degradation process and exact anatomical implant location, thus hindering accurate assessment of degradation-regeneration alignment [14]. Therefore, there is a need to design hydrogels that could be directly imaged to not only accurately localize, but also noninvasively monitor tissue regeneration while continuously tracking the degradation process of the hydrogel scaffold in situ [15].

MRI is one of the most commonly used non-invasive imaging modalities in clinical practice, which provides diverse imaging sequences with superior spatial resolution and high sensitivity to soft tissues, offering effective real-time feedback on the in-situ state of hydrogel [16]. Contrast agents (CAs) are frequently employed to enhance diagnostic accuracy by improving the image signal-to-noise ratio (SNR) [17]. However, commonly used MRI CAs are low molecular chelates based on Gd (III), present several disadvantages: low tissue specificity, rapid metabolism, high background noise, and a narrow imaging window [18]. Additionally, their low molecular weight ligands decrease the contrast agent’s relaxation rate, necessitating increased dosage and elevating the risk of long-term toxicity, such as in chronic kidney disease [19]. However, much of the current research focused on physically blending small-molecule contrast agents into biomaterial systems [20]. For instance, Alexandra Berdichevski and colleagues created an angiogenic hydrogel scaffold by physically blending Gd-DOTA with VEGF-loaded PEG fibrinogen, evaluating its degradation rate and angiogenic capability at various stages via MRI [21]. However, these physically blended imageable hydrogel scaffolds do not form molecular chains with the contrast agents, resulting in non-significant improvement in their T_1_ relaxivity. As a result, they were unable to produce high SNR MR images while minimizing CAs usage. Therefore, combining MRI CAs with macromolecular chains to design a CA-integrated hydrogel scaffold could simultaneously achieve the goals of high imaging efficiency and low toxicity, and enable in vivo, real-time monitoring of scaffolds and local tissue repair processes by MRI [22, 23].

**Scheme 1:**
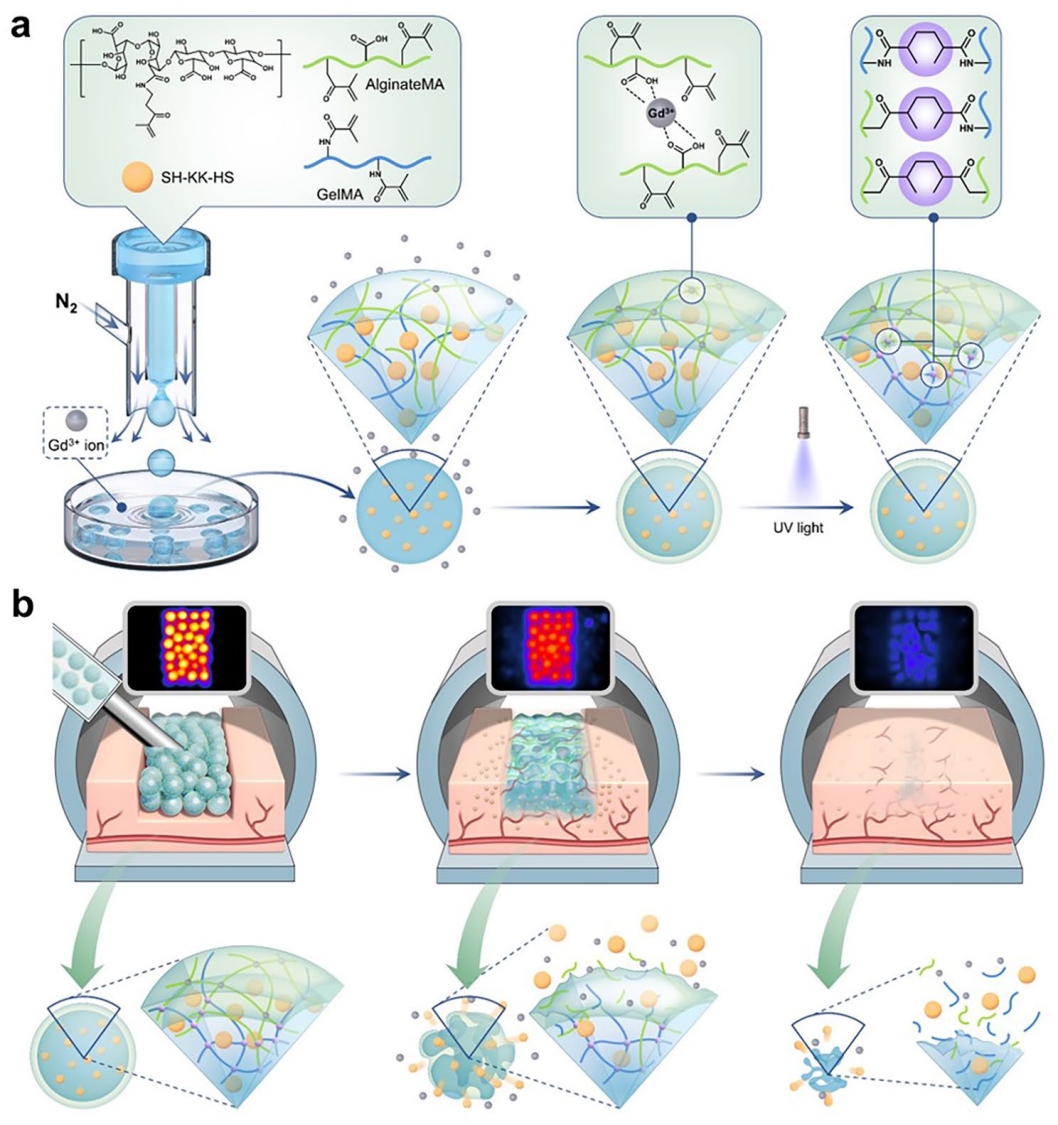
Illustration of the MRI-visible double-network hydrogel microsphere for dynamic monitor of soft tissue regeneration. **a**) Using gas shearing technology fabricating the G-AlgMA HMs with uniform size. 365 nm ultraviolet light was then used to further crosslink the network. **b**) Tissue regeneration leads to hydrogel network breakage and Gd (III) release, in the meantime, using MRI to monitor this dynamic process

In this study, inspired by the covalent cross-linking of sodium alginate with metal ions, HMs were fabricated using Gd (III) as a cross-linking agent via an air-flow-controlled platform. Methacrylated sodium alginate (AlgMA) blended with angiogenic peptides is used as the internal phase solution, with nitrogen as the external phase, replacing the traditional oil phase solution for cutting purposes. This approach constructs an MRI-visible, angiogenesis-promoting ‘dynamic monitoring-tissue repair’ integrated HMs. Additionally, these HMs were incorporated with methacrylated gelatin (GelMA) to improve biocompatibility and underwent further crosslinking with ultraviolet light (UV), resulting in dual-network HMs with approximately 200 μm in diameter (Scheme 1). In vitro, these dual-network MRI-visualized HMs demonstrate high T_1_ relaxivity, with an r_1_ value of 33.113M^− 1^s^− 1^, 3–4 times higher than that of the clinically used CAs, Magnevist®. In the meantime, their MRI signal decay rate correlates with the cell proliferation rate. They exhibited good biocompatibility and promoted endothelial cell vascularization under the influence of angiogenic peptides. In vivo, employing a rat soft tissue defect model, MRI precisely located these HMs at the implant site, and their degradation rate was inferred from signal changes. Additionally, extracellular matrix (ECM) deposition and neovascularization were observed, ultimately confirmed via histological staining.

## Results and discussion

### Fabrication and characterization

**Fig. 1:**
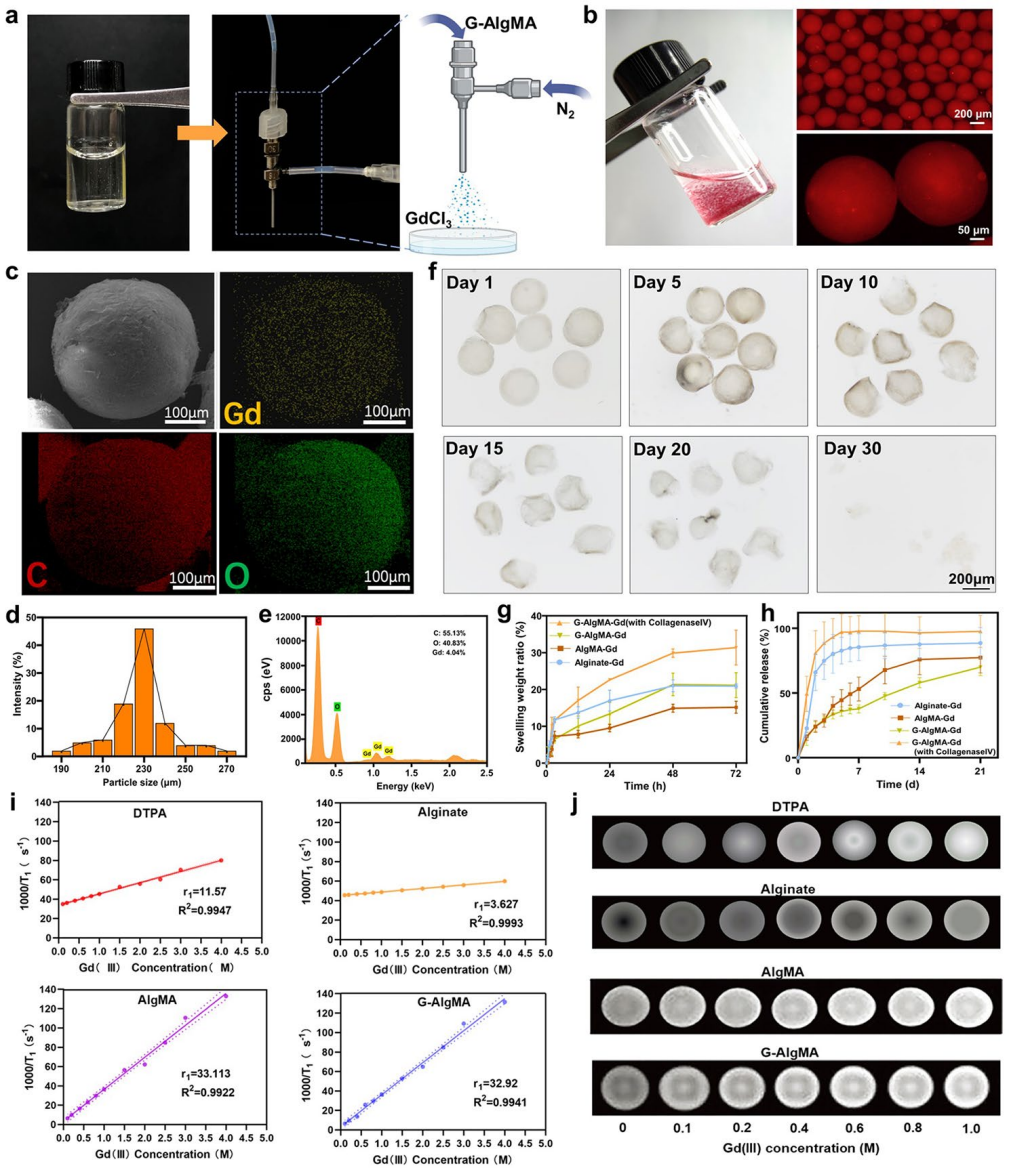
Synthesis of microspheres and enhancement characteristics of T_1_-weighted MRI in vitro. **a**) GelMA-AlgMA (left) was sheared into microspheres using gas-shearing device (middle). **b**) General view (left) and fluorescence microscope image of the G-AlgMA HMs labeled with Rhodamine, Scale bar 200 μm. **c**) SEM images and elemental mapping images (Gd, C, O) of G-AlgMA HMs. **d**) The size distribution of the microspheres. **e**) EDS spectra of the microspheres. **f**) The morphological changes of G-AlgMA HMs observed under optical microscope during degradation experiments. **g**) The swelling ratio of microspheres. **h**)Release curves of Gd (III). **i**) Linear plots of the relaxation rate (1000/T_1_) versus the Gd (III) concentration. **j**) T_1_-weighted MRI images of DTPA-Gd, Alginate-Gd, AlgMA-Gd, G-AlgMA-Gd with different Gd(III) concentration

A large number of carboxyl groups are present along the sodium alginate chain, facilitating easy interaction of carboxyl groups with divalent cations (e.g., Ca^2+^ and Mg^2+^) to form hydrogels [24]. Further, when trivalent cations are added (e.g., Al^3+^ and Fe^3+^), they can interact with the three carboxylate groups to form a more compact network, thus generating metal–alginate hydrogels with high mechanical properties. Motivated by this phenomenon, we cross-linked the rare earth trivalent cation, Gd (III), which is used to make magnetic resonance CAs, with sodium alginate to fabricate gadolinium–alginate HMs for potential use in T_1_-weighted contrast-enhanced magnetic resonance imaging. We first modified methacrylic anhydride onto the long chains of sodium alginate (Fig. S1). Furthermore, an appropriate amount of GelMA was added into the hydrogel precursor solution to improve the cross-linking density and biocompatibility of the microspheres [25] (Fig. 1a). The gas flow control method is more suitable than the traditional microfluidic control to fabricate G-AlgMA HMs. This method offers the advantages of one-step cross-linking and the use of nitrogen, instead of the oil phase, to act as a cutting agent. At the same time, the microspheres produced by the uniform particle size and the yield are extremely large. In this experiment, the AlgMA-GelMA precursor solution was transformed into spheres through nitrogen cutting. These spheres, upon entering the diffusion phase, were cross-linked with Gd (III) to form stable HMs.

Then, under 365 nm ultraviolet irradiation, the GelMA and AlgMA inside the microspheres were further photo-cross-linked to obtain the final GelMA–AlgMA–Gd (III) HMs with a double-network cross-linking structure (Fig. 1b). The microspheres were homogeneous and poorly dispersed with a diameter of 228.6 ± 14.7 μm (Fig. 1b and d). The surface structure of the microspheres was observed by scanning electron microscopy (SEM), and the energy spectral distribution showed that Gd (III) were uniformly distributed on the surface of the microspheres. The quantitative analysis revealed that gadolinium elements accounted for about 4.04% of all elements present (Fig. 1c and e). The microspheres must exhibit a degradation rate that aligns with tissue regeneration and be highly biocompatible for subsequent use in tissue repair. This is essential for simultaneous noninvasive in vivo monitoring of material degradation and tissue repair. After soft tissue damage, the tissue repair needs to go through an inflammatory exudative phase, a capillarization phase, and a collagenoplastic phase. Correspondingly, hydrogel scaffolds with reparative properties undergo swelling and gradual degradation [26]. We first investigated the swelling characteristics of our fabricated HMs to investigate whether HMs conformed to the real process in vivo. Dual-network cross-linked HMs in 0.9% saline swelled to 20% of their original mass within 48 h and then reached equilibrium (Fig. 1g), followed by a gradual release of Gd (III) during degradation of the hydrogel network. Inductively coupled plasma-mass spectrometry (ICP-MS) experiments revealed that single-ion cross-linked microspheres (Alginate-Gd HMs) exhibited the issue of an abrupt release of Gd (III), with Gd (III) almost disappearing from the network after 1 week. This clearly hampered the goal of achieving long-term anaplasticity and is not conducive to achieving long-term noninvasive MRI monitoring. On the contrary, the dual-network cross-linked GelMA–AlgMA–Gd microspheres (G-AlgMA HMs) could release Gd (III) slowly and gradually over time. The slow-release of Gd (III) was observed until day 21, indicating an appropriate degradation rate that could meet the needs for long-term in vivo monitoring. The degradation rate of the gelatin-incorporated HMs was significantly accelerated when type IV collagenase was added to 0.9% saline to more realistically simulate the in vivo environment, indicating their remarkable biocompatibility (Fig. 1h). Degradation rate of microspheres was controlled by adjusting the amount of collagenase. The degradation of the structure of the HMs was corresponding to the release of Gd (III). This degradation was observed by optical microscopy in the G-AlgMA HMs on the 30th day, indicating almost complete degradation of the structure (Fig. 1f). This finding aligned with the findings from the ICP-MS results. We investigated the longitudinal relaxation rate in vitro using an MRI machine to explore the potential of HMs as an MRI imaging platform [27]. As shown in the MRI image in Fig. 1j, the T_1_ signal intensity of Alginate-Gd microspheres and G-AlgMA microspheres enhanced with increasing Gd (III) concentration and exhibited similar signal intensities. However, Gd-DTPA, which is commonly used in clinical practice, did not show significant signal changes in the experimental concentration range. The signal intensity of HMs was greater than that of clinically used Gd-DTPA at the same Gd (III) concentration. Also, the r_1_ value of G-AlgMA HMs was 32.92 mM^− 1^s^− 1^, whereas the r_1_ value of Gd-DTPA was 11.57 mM^− 1^s^− 1^ (Fig. 1i). These results suggested that synthetic HMs could monitor the extent of tissue repair in T_1_-weighted images more effectively than commercial contrast agents. The rotational correlation time and water exchange rate are the main factors affecting the r_1_ value of the contrast agent. The slower the rotational correlation, the higher the water exchange rate of the contrast agent, resulting in a higher r_1_ value [28]. The three-dimensional structural features possessed by HMs allowed for increased exchange efficiency of water molecules. Further, the addition of alginate increased the molecular weight of the Gd (III) chelate, resulting in a slower rotational correlation. These two features acted synergistically to enhance the T_1_ signal intensity of G-AlgMA HMs. Also, although an increase in the molecular weight of alginate led to a slower rotational correlation accompanied by a higher r_1_ value, the correlation between the r_1_ value and the molecular weight was nonlinear. A molecular weight of 2000 was selected in our experiments, and subsequent exploration of the T_1_ imaging efficacy of different molecular weights of alginate chelated with Gd (III) was needed.

### Biocompatibility of HMs

**Fig. 2:**
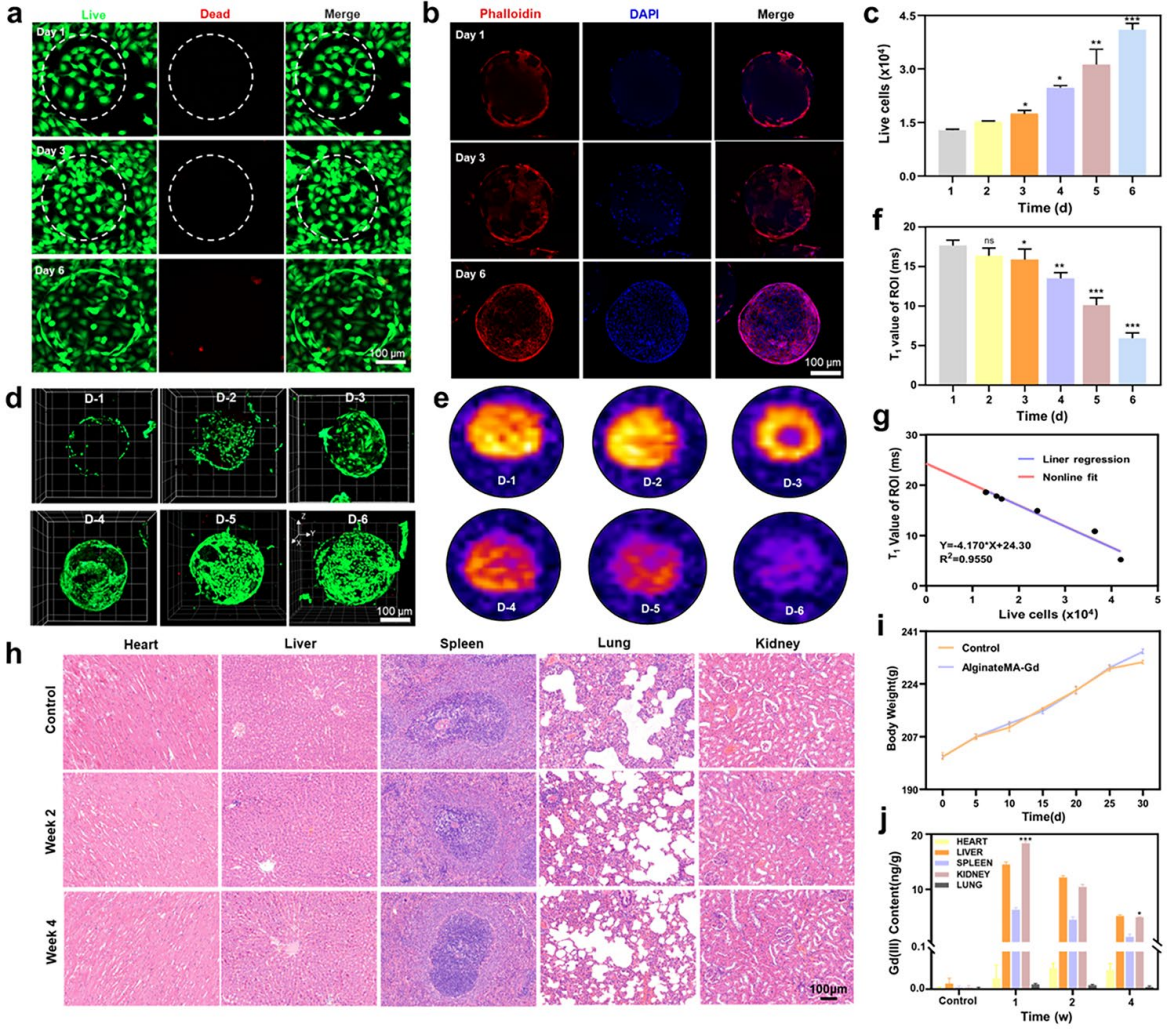
Biocompatibility of G-AlgMA HMs and in vitro MRI signal analysis. **a**) Live(green)/Dead(red) fluorescence results of G-AlgMA HMs on 1、3、6 days. **b**) Cytoskeleton staining of the HUVECs co-cultured with G-AlgMA HMs on 1、3、6 days. **c**) Viable cell count from the Live/Dead staining assay. **d**) The z-stack fluorescent images of cells adhered on HMs. **e**) T_1_-weighted MRI images of G-AlgMA HMs with adhered HUVECs on 1–6 days. **f**) The T_1_ value of G-AlgMA HMs with adhered HUVECs. **g**) Correlation of T_1_ value and viable cell number of G-AlgMA HMs on specific day (liner regression showed the Correlation coefficient R²=0.955. **h**) HE-stained tissues of heart, liver, spleen, lung and kidney of SD rats provided with saline (control) or G-AlgMA HMs in different time points. **i**) Body weight observed during 30 days for the control (saline) and G-AlgMA HMS groups. **j**) Biodistribution of Gd (III) in the rats obtained at day 1, 2 and 4 after treated with saline (control) and G-AlgMA HMs.

As contrast agents containing Gd (III) chelates for clinical applications, most MRI agents reach normal tissues after injection. The potential metabolic toxicity of these agents to normal cells and organs arises due to the possibility of Gd (III) production from chelate dissociation. In addition, as a scaffold material for tissue repair, HMs should also provide a good simulated physiological environment for cell adhesion and proliferation. Therefore, this study explored the biocompatibility of G-AlgMA HMs. First, human umbilical vein endothelial cells (HUVECs) were used to explore the biocompatibility of microspheres in vitro. The HMs and cells were co-cultured in two and three dimensions, respectively, and the effects of HMs on cell viability and proliferation were assessed by live cell staining and cell counting kit-8 (CCK-8) assays (Fig. 2a and S2). As shown in Fig. 2c, a vast majority of cells remained viable during 6 days of incubation and showed significant proliferation on days 5 and 6. We performed 3D confocal imaging of HMs in different stages and found that HUVECs had significant proliferation in the 3D structure (Fig. 2d). These results suggested that G-AlgMA HMs had good cell biocompatibility and provided a suitable growth environment for HUVECs. In addition, we also investigated the in vivo safety of G-AlgMA HMs and the metabolism of Gd (III). Sprague–Dawley (SD) rats with rectus abdominis muscle defects were filled with HMs and injected with saline (control group) at the defect site. Then, the body weights of rats in the experimental and control groups were measured on the 30th postoperative day. No significant differences were found (Fig. 2i), and no deaths of rats were observed. On day 30, the vital organs of experimental and control rats were subjected to hematoxylin and eosin (H&E) staining of tissue sections, and no lesions were found in the vital organs of rats filled with HMs (Fig. 2h). In general, the large and prolonged retention of Gd-based HMs in the body may lead to the development of undesirable diseases induced by Gd (III). To further evaluate the metabolism of G-AlgMA HMs in vivo, Gd (III) concentrations were measured in major organs of mice in weeks 1, 2, and 4. As shown in Fig. 2j, relatively low levels of Gd accumulated in the major organs of the rat and relatively high levels were observed in the kidneys, liver, and spleen, suggesting that the degradation products of the HMs were mainly metabolized by those organs. Also, the Gd (III) concentration in the kidney, liver, and spleen gradually decreased over time to 2.56 ng/g in week 4. Rapid metabolism may damage the kidney, whereas slow metabolism leads to the accumulation of heavy metals in the body. Therefore, the slow-release effect of the HMs avoids contrast-induced acute kidney injury. However, even in week 4, when the Gd (III) concentration was no longer damaging to the organism, we still had to be vigilant about the in vivo deposition of Gd (III), for example, in brain parenchyma deposition. Lower concentrations were used to achieve superior contrast and meet diagnostic needs while ensuring biosafety.

### Correlation between changes in MR signaling and cell number

We first explored the consistency of the magnetic resonance T_1_ signal values with the changes in cell number in vitro to ultimately monitor the metabolism of localized HMs in an in vivo model using the changes in magnetic resonance signals. The HMs were co-cultured with HUVECs in three dimensions (3D). Also, the cell live–dead staining and in vitro MRI were used to explore the correlation between the number of cells in the HMs and their corresponding T_1_ signal values. As shown in Fig. 2d, a significant increase in the number of live cells was noted over time. In contrast, the T_1_ signal value of the corresponding HMs decreased accordingly (Fig. 2e and f). The correlation analysis between the number of cells and the T_1_ signal value at the same time point revealed that the correlation coefficient R^2^ of the two reached 0.955 (*P* < 0.01), indicating an obvious correlation (Fig. 2g). This suggested that we could determine the number of cells in the 3D structure of HMs using the T_1_ signal value of the HMs, which laid the foundation for the subsequent in vivo monitoring. As shown in Fig. 2b, while proliferating, the morphology of the cytoskeleton remained normal. The proliferation of cells in the 3D structure of the hydrogel destroyed the original cross-linking between the macromolecular chains, which led to the dissociation of a part of the Gd (III) chelated on the molecular chains of alginate. The free Gd (III) was not effective in shortening the longitudinal relaxation time, that is, they did not have the T_1_ signal enhancement efficacy. This led to the reduction of the T_1_ signal of the HMs. However, the chelation of alginate and Gd (III), besides G-AlgMA HMS, also had the presence of photo-crosslinking structures between AlgMA and GelMA. Hence, the dissociation of the photo-cross-linking sites due to cell proliferation did not result in a change in the T_1_ signal value of the HMs. Therefore, the sensitivity of the T_1_ signal value of the HMs to cell proliferation needed further exploration.

### Monitoring in vivo degradation by MRI

**Fig. 3:**
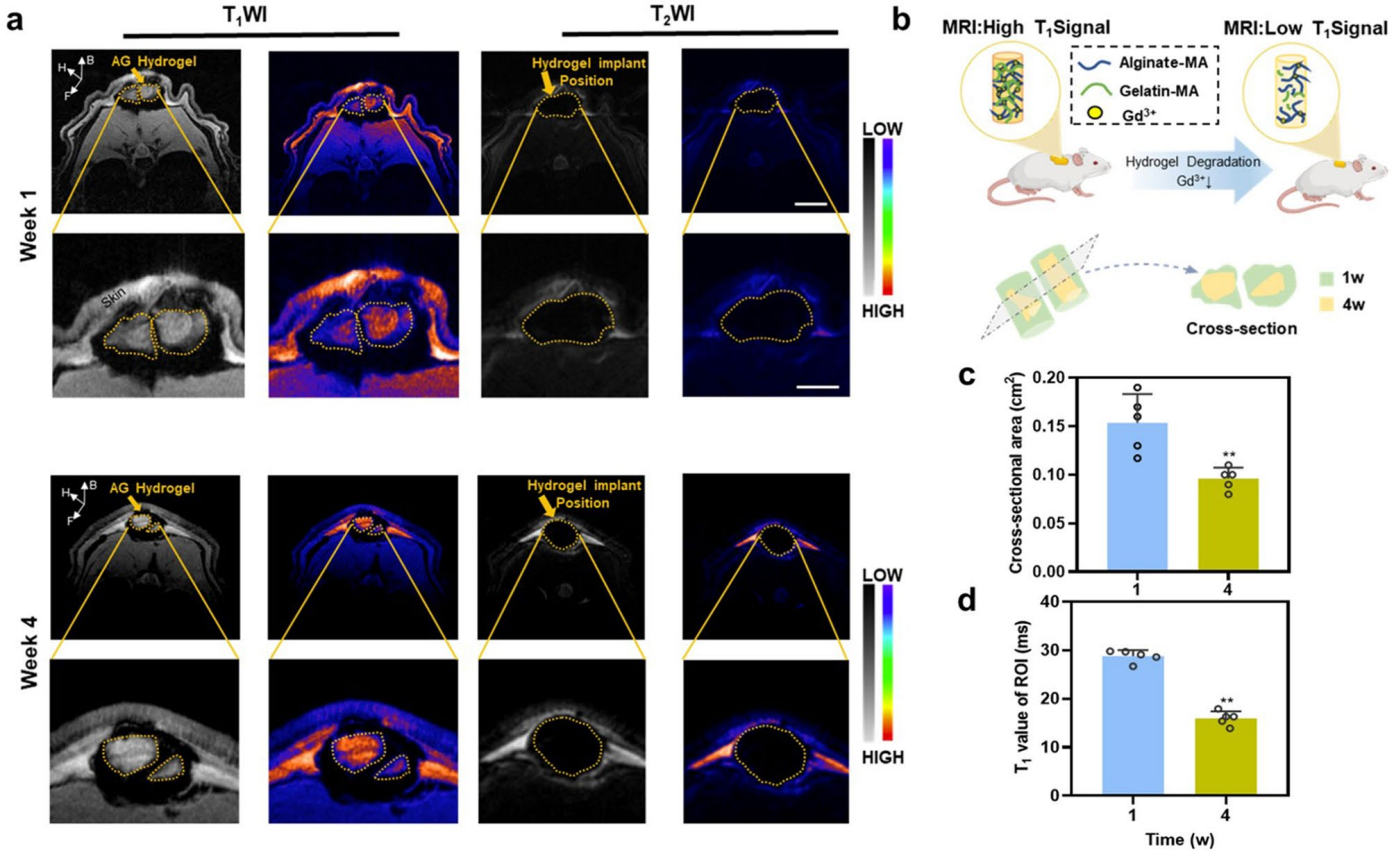
MRI image analysis of degradation in vivo. **a**) Representative T_1_WI and T_2_WI axial plane MRI images of cylinder consisted of AG hydrogel on 1 and 4 weeks. Arrows indicate the Gd-labeled implants. **b**) Schematic diagram of relationship between the concentration of Gd (III) ions and the network of AG hydrogel. **c**) Cross-sectional area of cylinder consisted of AG hydrogel calculated on T_1_WI axial plane MRI images. **d**) T_1_ values of the region of interest (ROI, dotted line areas showed on MRI images)

In this section, cylindrical hydrogels made of AlgMA and GelMA mixed in a 3:1 ratio and then cross-linked with Gd (III) (AG hydrogel) were used to investigate the relationship between the degree of degradation in vivo and the signals of the MR images instead of HMs in order to localize the subcutaneously implanted hydrogel better under MRI [29]. MRI T_1_-weighted and T_2_-weighted scanning of the implant sites was performed in the first and fourth weeks after implantation, respectively. When the implant is degraded in the organism and the macromolecular chain is broken, the Gd (III) cross-linked in the HMs are dissociated from the macromolecular chain. The free Gd (III) do not have an MR signal enhancement effect, and hence the MRI signal is correspondingly reduced at this time [30]. Based on the aforementioned principle, we attempted to use the change in the value of the MRI signal to infer the degree of degradation of the implant in vivo (Fig. 3b). In animals, the implant itself is a macromolecular hydrogel structure. Hence, the inherent rotation frequency of the molecule is relatively low, much lower than its Larmor frequency; therefore, the T_1_ value is relatively long [31]. The addition of gadolinium provides the necessary contrast between the implant and the surrounding tissues. This enables a clearer observation of the degradation of the implant in vivo, particularly through the T_1_-weighted image. The corresponding T_2_-weighted image could provide limited information about implant degradation (Fig. 3a). Axial MRI T_1_-weighted images revealed the degradation of the cylindrical implant composed of GelMA-AlgMA-Gd in rats.

First, the implant remained stationary in its position without moving throughout the observation period (Fig, 3a, yellow-dotted line). Second, the T_1_-weighted axial images showed that the cross-sectional area of the implant on the back of the rats was reduced by 0.057 ± 0.028 cm² in the fourth week compared with that in the first week (*P* < 0.01) (Fig. 3c). At the same time, our measurements of the T_1_ values at the implant site revealed that the T_1_ signal value of the ROI was reduced in the fourth week compared with the first week by 12.820 ± 2.532 ms (*P* < 0.01) (Fig. 3d). The correlation analysis was performed on the change in cross-sectional area (△area) and the change in T_1_ signal value (△T_1_ value), revealing a more obvious correlation between the two (R² = 0.833, *P* = 0.031) (Fig. S4). It indicated that the change in T_1_ value in MRI reflected the degree of degradation of in vivo implants to a certain extent and had good sensitivity area under the curve (AUC) = 0.91. The gold standard for assessing the real in vivo processes is the pathological Sect. [32]. Alexandra Berdichevski et al. conducted a study comparing MRI and pathological sections to investigate the degradation rate of implants with different shapes in vivo, concluding that the signal change of MRI was basically the same as the results of pathological Sect. [33]. In our experiments, the in vivo degradation was investigated only by comparing the degree of change in the signal value of the implant with the cross-sectional area on MRI scans. Also, the comparison of pathological sections was lacking, which could not exclude the shedding of Gd (III) for reasons other than the degradation of the macromolecular chains. This needs to be improved in the subsequent experiments.

### Rat abdominal soft tissue defects,  noninvasive monitoring of tissue repair and degradation of HMs using MRI

**Fig. 4:**
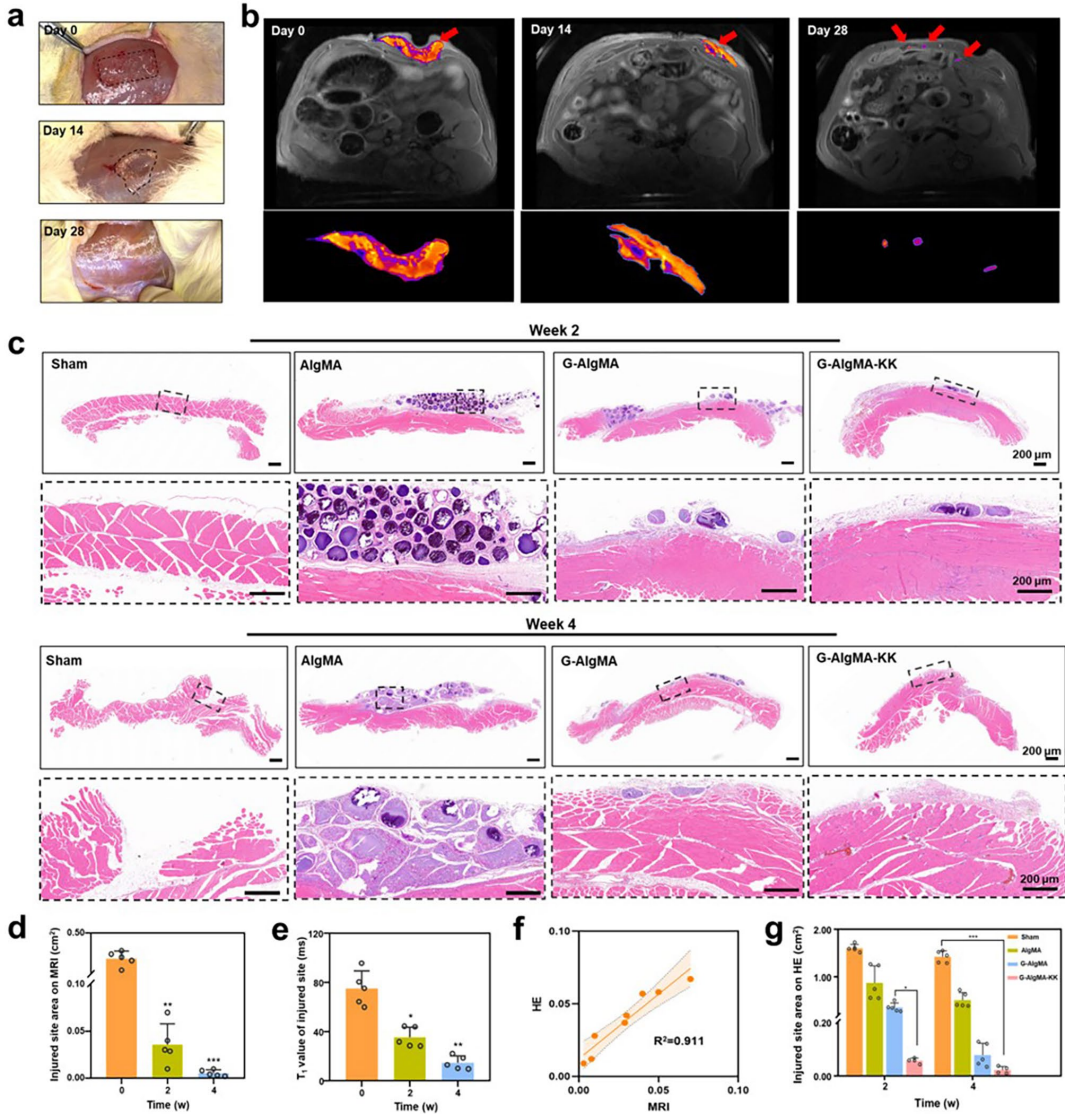
In vivo dynamic monitor of soft tissue regeneration via MRI and histological analysis of tissue repair. **a**) Representative photographs of injured site with G-AlgMAs implants on 0, 14, 28 days. **b**) T_1_-weighted abdominal axial MRI images of injured site with G-AlgMAs implants on 0, 14, 28 days. Arrows indicate the Gd-labeled implants. **c**) Representative images of H&E staining from each group. **d**) Area and e) T_1_ value of injured site via MRI images at different time points. **f**) Correlation between T_1_ value of MRI images and injured site area of H&E staining images on specific day(liner regression showed the Correlation coefficient R²=0.911). **g**) Injured site area on H&E images of each group on 2 and 4 weeks

Experiments above have examined the relaxation rate and biocompatibility of HMs in vitro and the feasibility of using MRI to monitor the degree of material degradation in vivo. Subsequently, it was essential to examine the feasibility of HMs in an in vivo disease model to monitor the degree of material degradation and tissue repair at the defect site [34]. We chose the abdominal muscle defect model for validation because this model was close to the subcutis, which was easier to observe and locate under MRI compared with deep tissues. Also, the range of the abdominal muscle defect model was in line with the resolution of the image that could be observed using 7.0-T MRI, which was more conducive for us to determine the degree of tissue repair in the MRI scans [35]. AS the purpose of animal magnetic resonance was to verify whether the signal of the MRI could reflect the degradation of the HMs in vivo, along with monitoring the regeneration of local tissues. For this purpose, the group with the best tissue repair efficacy, GelMA-AlgMA-Gd with K2 (SL)6 K2 peptide (G-Alg-KK), was selected for MRI study in the experiment. As shown in Fig. 4a, a 3 × 1 defect was created in the abdominal muscle of SD rats, with a depth that did not exceed the peritoneum. This defect site was filled with our synthesized microspheres, and the HMs were fixed using “bio-glue“ [36]. Abdominal MRI scans were performed on days 0, 14, and 28 after the surgery. The signal at the rectus abdominis defect was significantly enhanced in the axial image on day 0 (Fig. 4b, red arrow). This corresponded to the location of the filling material after modeling (Fig. 4a, black-dashed line). MRI scanning of the same abdominal region was performed again on the same patient 1 week after the modeling. In MRI, we regarded the region of localized signal enhancement as the defective area. After selecting the region of interest (ROI), the image processing software Matlab was used to calculate the area of ROIs. In the axial T_1_-weighted image, the signal at the abdominal defect site remained elevated compared with the surrounding rectus abdominis muscle. However, its signal intensity and the extent of the injury decreased compared with that observed on day 0. The quantitative analyses indicated that the changes had values of 0.306 ± 0.032 cm² and 39.820 ± 9.800 ms, respectively (*P* < 0.001) (Fig. 4b, red arrow, black-dashed line, and Fig. 4d and e), which was also confirmed by actual autopsy. The enhanced signal was barely visible on the MRI scans by week 4. We hypothesized that the filled HMs had almost completely degraded by this time. Also, the initially elevated signal at the defect site was replaced by a normal rectus abdominis muscle signal, as confirmed during autopsy. The observed scenario closely aligned with the findings in the MRI images (Fig. 4a and b). We performed H&E staining on the tissue sections to further validate the feasibility and sensitivity of MRI signals for detecting tissue repair (Fig. 4c). Also, we fitted the regeneration range in the sections with the MRI signal intensity, revealing a significant correlation between the two (R² = 0.911, *P* < 0.01) (Fig. 4f). This finding led to the conclusion that the degradation of the repair material in vivo and the regeneration of the tissue could be monitored in real time by the changes within the region of interest (ROI) in the MRI T_1_-weighted image.

### Histological analysis of repairing soft tissue defects

Angiogenesis plays a crucial role in tissue regeneration, emphasizing the need for scaffold materials in soft tissue repair to not only exhibit good biocompatibility and provide a three-dimensional environment conducive to cell growth but also promote angiogenesis [37]. Therefore, we incorporated the pro-angiogenic peptide K2 (SL)6 K2 (KK) into G-AlgMA HMs and first evaluated its ability to promote angiogenesis in vitro [38]. As shown in Figure S3, HUVECs exhibited more branches and longer tube lengths than controls when cultured in KK-added HMs. Moreover, the vasculogenic capacity exhibited by HUVECs was continuously optimized with the increase in peptide KK addition (10, 50, and 100 U), which might be attributed to the stimulation of vascular endothelial growth factor (VEGF) expression by adding KK peptide (Fig. S3). However, when KK exceeded 100 U, both branching and tube length decreased. The substantial secretion of VEGF in the early stage facilitates the recruitment, proliferation, and differentiation of endothelial cells to form endothelial tube-like structures [39, 40]. However, if a large amount is accumulated for a long time, nonfunctional blood vessels are formed or even the proliferation of endothelial cells is inhibited. This may be the reason why excessive vasculogenic polypeptide KK inhibits the tube-forming differentiation of endothelial cells. Based on the aforementioned findings, we added 100-U KK peptide into gelatin–gadolinium–alginate HMs for subsequent animal experiments.

**Fig. 5:**
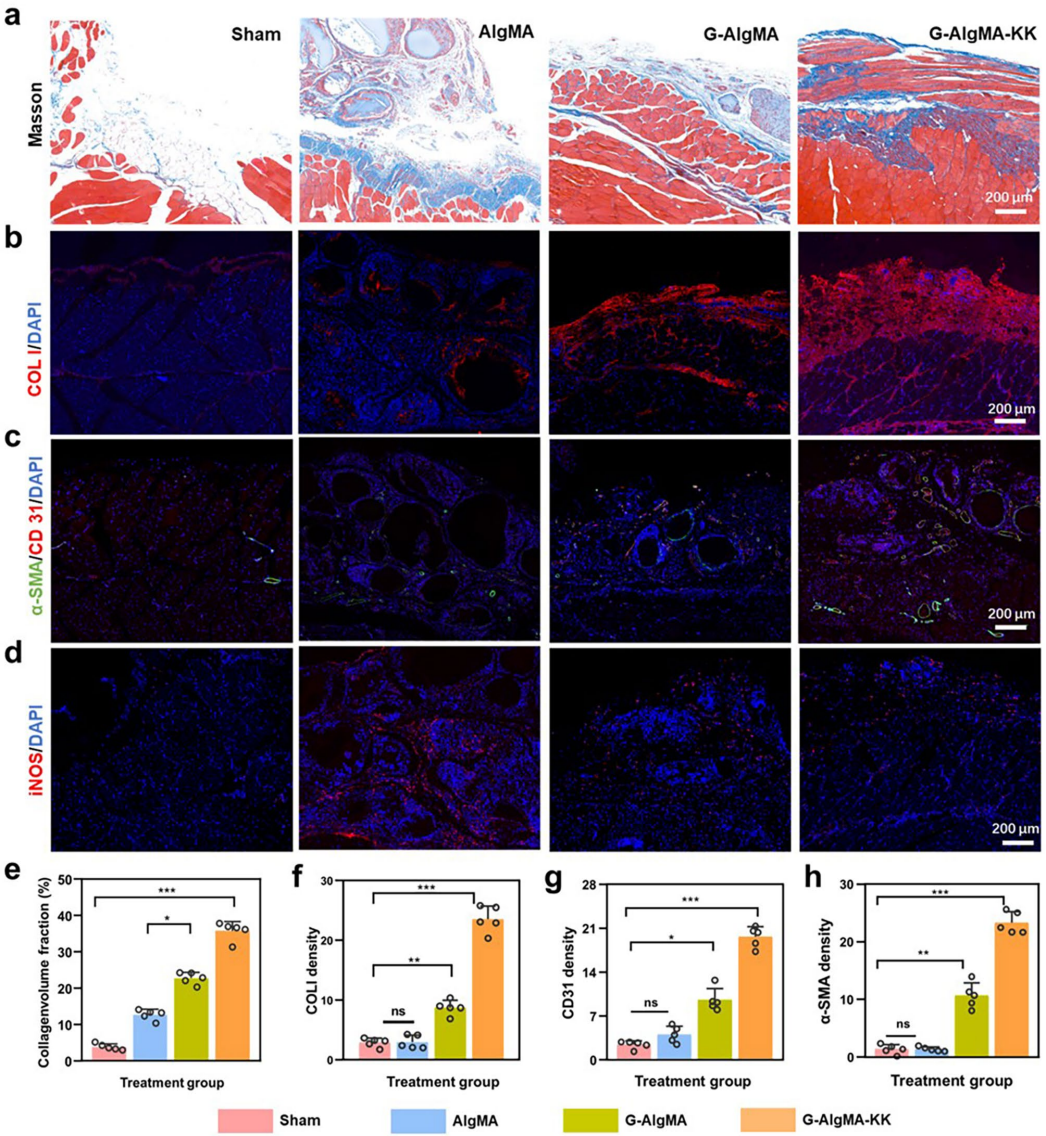
G-AlgMA HMs accelerate soft tissue regeneration after 4 weeks of operation. **a**) Representative images of Masson staining from each group. **b**) Immunofluorescence analysis of COL I, **c**) α-SMA and CD 31, **d**) iNOS in regenerate tissue. **e**) The quantification of collagen deposition. **f**, **g**, **h**) The semi-quantitative analysis of immunofluorescence of COL I, CD 31, α-SMA

All experimental animals were randomly categorized into Sham, AlgMA-Gd HMs (AlgMA), GelMA-Gd-AlgMA HMs (G-AlgMA), and GelMA-Gd-AlgMA HMs with KK peptide (G-AlgMA-KK) groups. The rats in the sham group were treated with saline, whereas the rats in the other groups were treated with the respective materials. The HE was also carried out to evaluate the biological mechanism of wound healing, and the results showed that the defect area in the G-AlgMA and G-AlgMA-KK groups achieved re-epithelization and granulation tissue formation during the remodeling stage, while the wounds in the Sham and AlgMA groups had delayed healing. In general, we determine the extent of the injured area based on the degree of wound healing, wound length, and granulation tissue thickness in each group. The trend in the G-AlgMA-KK group was the most pronounced among the groups. Compared with the G-AlgMA group, the G-AlgMA-KK group showed more tubular angiogenesis in both weeks 1 and 4, as evidenced by a significant increase in the expression of CD31 and α-smooth muscle actin (α-SMA) (*P* < 0.001) (Fig. 5c, g and h, and S5); more ECM deposition, as evidenced by a significant increase in the expression of collagen I (Col I) (*P* < 0.001) (Fig. 5c and g, and 5h) [41]; less M1-type macrophage invasion, as evidenced by a significant decrease in inducible nitric oxide synthase (iNOS) expression [42] (*P* < 0.001) (Fig. 5d and S5). This was attributed to the addition of the KK peptide, which led to the formation of more blood vessels in the defect site. The H&E and Masson staining results in the G-AlgMA-KK group showed essentially complete wound healing with more muscle fiber components and complete degradation of the HMs by week 4 (Figs. 4g and 5a). Quantitative analysis of the collagen-volumn fraction demonstrated the same result (Fig. 5e). The degradation performance of AlgMA group was poor. Therefore, we added GelMA to improve the degradation performance of the HMs and enhance the biocompatibility. This modification was previously validated in in vitro experiments. We further verified it in our in vivo experiments. In the AlgMA group, some HMs were still left in the defect sites in week 4. Also, a significant difference was found in the area of the defects compared with that in the G-AlgMA group (*P* < 0.01). Additionally, the muscle fiber content, ECM deposition, and the degree of neovascularization were all significantly different from those in the G-AlgMA group (*P* < 0.001). This indicated that the addition of GelMA also promoted the degradation of HMs in vivo, highlighting the significance of the gradual degradation of microspheres as a necessary condition for tissue repair at the defect site. All of the cell lineages need to contribute to the repair process and a variety of mechanical and chemical signals trigger these orchestrated cell behaviours. Many of these signals come from inflammatory cells, which themselves are drawn to the wound by damage signals and by signals from the pathogens that they have been primarily recruited to stave off. The dominant pro-angiogenic factor during wound healing is VEGFA, which is released by wound edge epithelial cells and macrophages [43]. We hypothesized that the G-AlgMAs without KK peptide stimulate local vascular regeneration further promotes collagen deposition by initially attracting peripheral macrophages around the injury site adhering to the interstitial space of the microspheres and promoting VEGFA secretion. Based on these results, we concluded that gelatin–gadolinium–alginate microspheres, when incorporated with the vasculogenic peptide KK, could promote vascular regeneration in situ with degradation properties aligning with the rate of tissue regeneration. This provided a robust 3D platform for reconstructing soft tissue defects.

## Conclusion

In summary, we designed a repair-monitoring integrated dual-network hydrogel microsphere for noninvasive dynamic monitoring of soft tissue repair and regeneration. The rare earth ion Gd (III) was chelated with alginate using an air-flow-controlled method, and simultaneous monitoring of material degradation and tissue repair was achieved by MRI. The longitudinal relaxation rate of the microspheres was three times higher than that of the clinically used MRI T_1_-weighted CAs, Gd-DTPA, which gave better SNR with less dose and reduced the potential toxicity associated with the in vivo deposition of rare-earth metal elements. By coupling the Gd(III) shedding caused by the decrosslinking of the hydrogel network with cell proliferation and in situ tissue regeneration, the change of magnetic resonance signals can not only determine cell proliferation in vitro, but also monitor the in situ regeneration of rectus abdominis muscle in vivo, which replaces the traditional pathology slides, overcomes its invasive and lagging shortcomings, and shows the dynamic process of biomaterials and in vivo tissues in a non-invasive and real-time way, and accelerates the development of biological regenerative materials into clinical practice.

## Material and method

### Synthesis of G-AlgMA HMs

Alginate-MA was synthesized according to previous description. In brief, alginic acid sodium (Sigma-Aldrich) was dissolved in carbonate bicarbonate buffer (0.1 m) under stirring. Different volumes of methacrylic anhydride (Aladdin) were added dropwise to the alginic acid sodium solution and allowed to react for 3 h. The reaction was stopped by adding excessive PBS. The diluted mixture was dialyzed in deionized water for 1 w and the purified sample was then freeze-dried for 3d to form a white foam. Air-flow controlled shear equipment was used to fabricate microspheres [44]. Alginate-MA (1% w/v) and GelMA (10% w/v) were dissolved separately with an aqueous solution containing 5% photoinitiator (Alladin), and the two were mixed in a 3:1 ratio (3.25% w/v). The mixed aqueous solution was injected through a coaxial needle and cut into homogeneous droplets (0.4 L min^− 1^) under the shear force generated by nitrogen. The collection tank was an aqueous gadolinium chloride solution (GdCl_3_, 0.1 mol L^− 1^, Sigma-Aldrich), and Gd (III) was cross-linked with Alginate-MA to form hydrogel microspheres. The microspheres were collected and washed twice with 0.9% NaCl solution before irradiating the microspheres with a 365 nm light source for 5 min to further photo-crosslink the microspheres. Finally, the dual network cross-linked HMs were collected and stored at 4 ℃. The whole process was operated in a sterile environment.

### T_1_ relaxation rate

The T_1_ relaxation rate of HMs was evaluated at room temperature using 7.0 T MRI (Bruker Science) with an 8-channel phased array coil. The Gd (III) concentration in different groups was varied by changing the concentration of GdCl_3_ in the collection tank when using airflow-controlled fabricated microspheres, and the clinically used MRI contrast agent Gd-DTPA was used as a control. To obtain T_1_-weighted images, we used the following sequence parameters. A curve of 1000/relaxation time (1000/T_1_, s^− 1^) versus Gd (III) concentration was constructed to obtain the slope as the T_1_ relaxation value (r_1_) for the corresponding group. The images were analyzed pixel by pixel by MATLAB. We used the following parameters and sequences: Time of inversion (TI) = 50,100, 200, 500, 800, 1200, 1500 ms; echo time (TE) = 8 ms; repetition time (TR) = 5000 ms; matrix size = 128 × 256; number of excitations = 3; echo sequence length = 8. Constructing a curve of 1/relaxation time (1/T1, s^− 1^) versus Gd (III) concentration, the relaxation value (r_1_) was obtained as its slope [45].

### Cell adhesion and in vitro MRI

HUVECs cells (ATCC) were centrifuged and resuspended and planted on the surface of GelMA-AlgMA-Gd microspheres in 24-well plates (0.4 μm pores, Corning). After 1, 2, 3, 4, 5 and 6 days of co-culture, live-dead staining and FITC/DAPI (Beyotime) staining were performed, and the number and morphology of the cells on the microspheres were observed under a confocal microscope. In order to further explore the relationship between the number of cells and the changes of MRI signals on the microspheres, we rinsed the HMs at the corresponding time points with 0.9% NaCl solution, collected them into 1.5 mL ep tubes (Corning), and scanned them with a 7.0T MRI machine with an 8-channel phased-array coil. And the images were processed with MATLAB to obtain quantitative signal values.

### In vivo experiment

A rat subperitoneal muscle defect model was used to verify the efficacy of HMs for tissue repair and the feasibility of real-time MRI monitoring of the whole process. The animal experiments have passed the ethical approval of Renji Hospital of Shanghai Jiaotong University School of Medicine. SD rats weighing 200 g were used for the experiments and cultured in a standard SPF environment. After anesthesia, at the rectus abdominis muscle of rats, the skin flap was lifted up, and the super muscular layer and fascial tissues (2 × 4 cm) were stretched in four directions with four hemostats for 1 min, and then the microspheres were filled in the 1 × 3 cm abdominal defect area [46]. The consistency between each experimental animal was ensured by the area of the injury site and the weight of the filled microspheres. In order to reduce the immune response, control the variables and better investigate the effect of microspheres on tissue repair, we fixed the microspheres on the soft tissues with bioadhesive and subsequently closed the skin with 4 − 0 sutures. All 20 rats were subsequently divided into 4 groups; Sham (treated with saline), AlgMA, G-AlgMA and G-AlgMA-KK (*n* = 5). Abdominal 7.0T MRI scans (Bruker Science) were performed on modeled rats. An 8-channel abdominal coil was used, supplemented by respiratory gating and ECG monitoring. The imaging protocol consisted of a T_1_-weighted spin echo and a T_2_-weighted turbo spin echo sequence. T_1_-weighted images were generated using a Fast Low Angle Shot (FLASH) sequence with parametres as follws: TE = 2.9 ms, TR = 400 ms, Flip Angle = 50, FOV = 300 mm×250mm^2^, slice interval = 1 mm, slices = 30, matrix = 192 × 160, resolution = 0.156 × 0.156 mm^2^, Average = 3. T_2_-weighted images were generated using a Tubro Rapid Acquisition with Relaxation Enhancement (RARE) sequence with parametres as follws: TE = 25 ms, TR = 3000 ms, FOV = 300 mm×250mm^2^, resolution = 0.117 × 0.117 mm^2^, slice interval = 1 mm, slices = 30, matrix = 256 × 214, Average = 3 [47]. The acquisition time is the same as the acquisition time for the other two data sets.

### Histological analysis

Abdominal muscle tissues of rats were taken at 4 and 8 weeks after surgery, and HE and Masson trichrome staining was used to evaluate the wound healing effect. Immunohistochemical staining was performed with rabbit polyclonal antibody (Abcam). COL I, CD31, α-SMA, and iNOS expression were quantified by ImageJ.

### Statistical analysis

Statistical data were processed and analyzed with GraphPad Prism 7.0 (GraphPad, USA) and SPSS software version 19.0 USA (IBM). In this case, quantitative data were expressed as mean ± standard deviation or median, and categorical data were expressed as numbers (percentages). One-way and multifactor ANOVA were used to compare differences between two or more groups. *p* < 0.05 was considered statistically significant.

### Electronic supplementary material

Below is the link to the electronic supplementary material.

Supplementary Material 1

## Data Availability

No datasets were generated or analysed during the current study.
