# Supplementary material for: Dynamic monitoring soft tissue healing via visualized Gd-crosslinked double network MRI microspheres
Source: J Nanobiotechnology. 2024 May 27;22:289. doi: 10.1186/s12951-024-02549-7 (PMC11129422; doi:10.1186/s12951-024-02549-7)
Supplement: Supplementary file 1 — Supplementary Material 1 [file 12951_2024_2549_MOESM1_ESM.docx]

**Supplemental Materials**

**
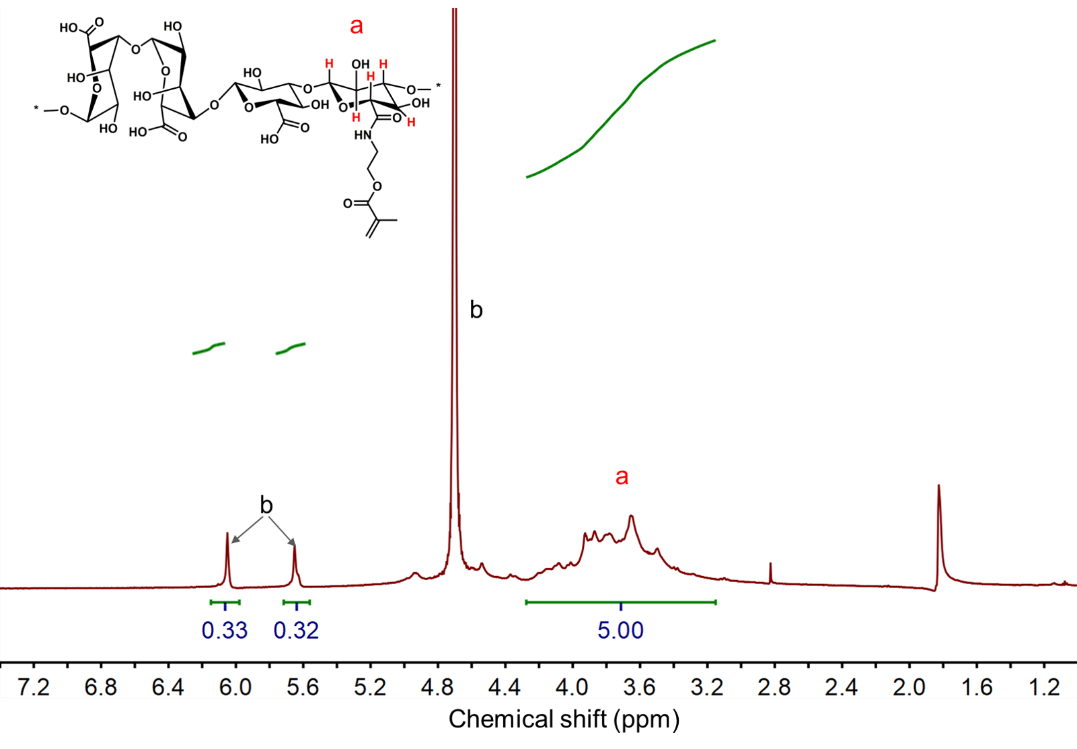
**

Figure S1: The substitution degree of the methacrylic anhydride group (≈32.5%) was determined by ^1^H NMR.


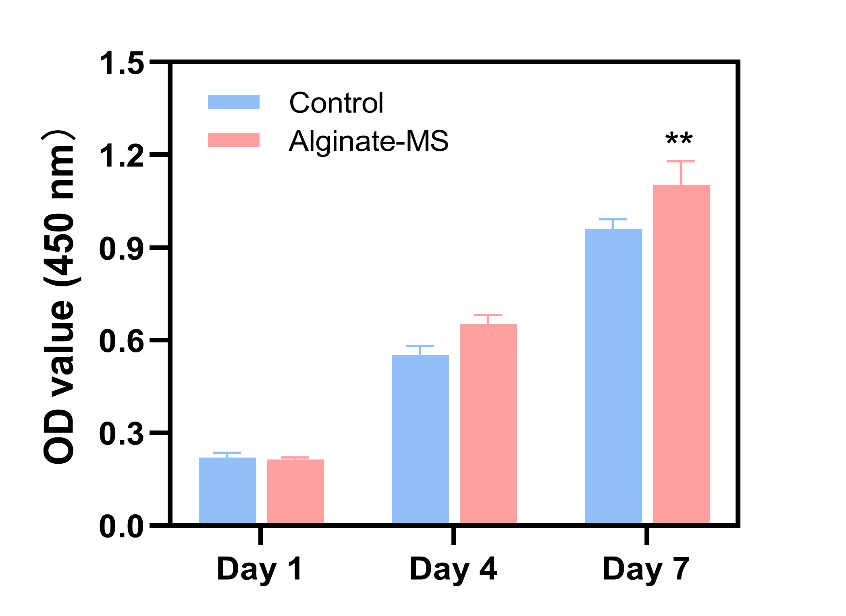


Figure S2: CCK-8 assay to detect cell viability. When co-cultured with microspheres, the number of cells increased significantly over time, and the number of cells was not significantly different from the control at all time points.


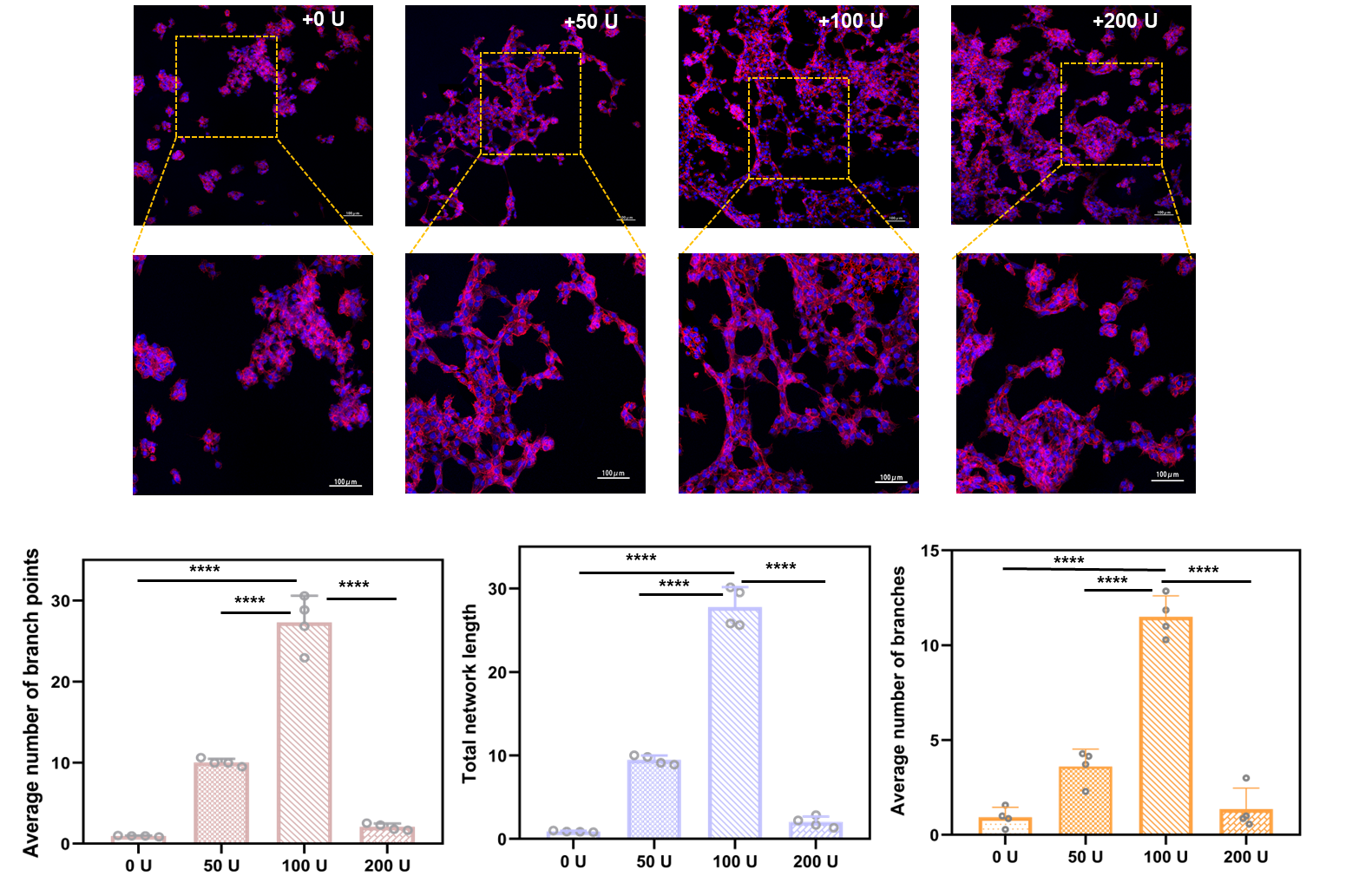


Figure S3: In vitro angiogenic assay: different peptide concentrations (0U, 50U, 100U, 200U) were selected to induce angiogenesis in HUVESs. The most obvious effect was observed at 100U, which was chosen for in vivo experiments.


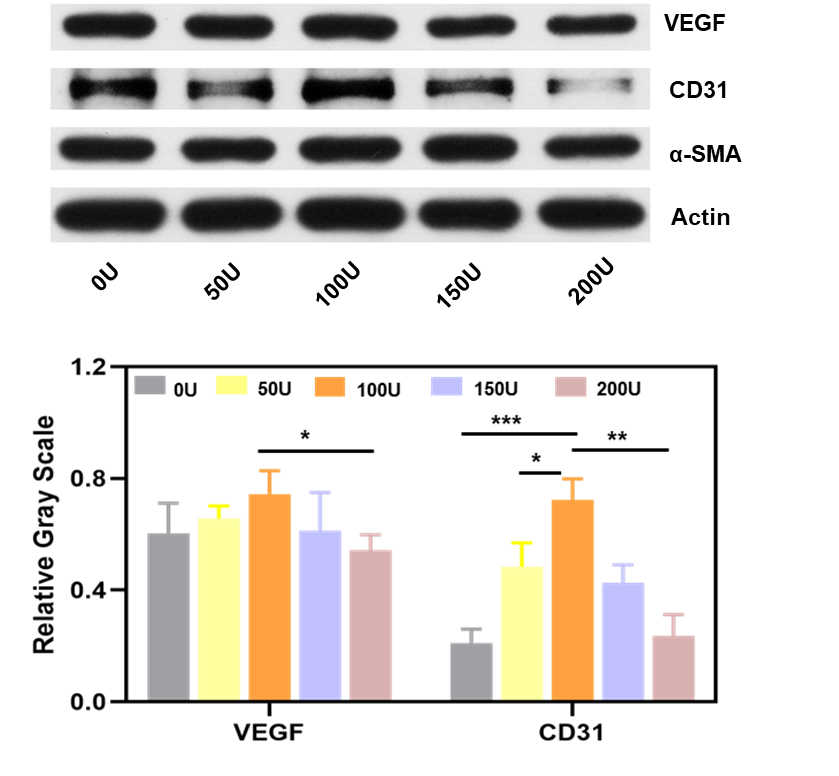


Figure S4: Quantitative analysis of angiogenesis-related proteins was performed using Western Blot. The most significant expression of CD31 and VEGF was observed at a peptide of 100 U, further indicating that 100 U is the optimal concentration.


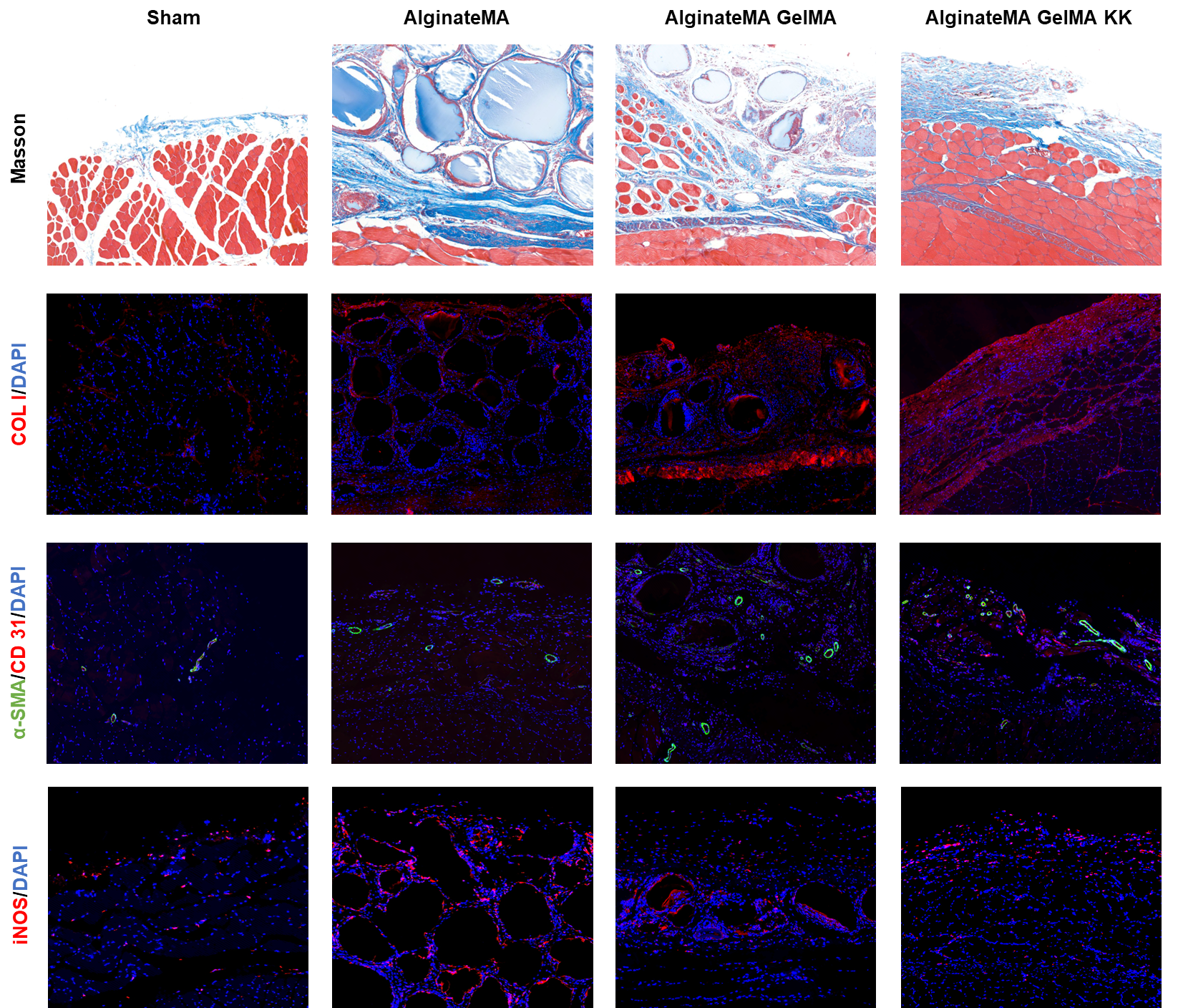


Figure S5: The histopathological section at the second week.
